# Machine learning for comprehensive prediction of high risk for Alzheimer’s disease based on chromatic pupilloperimetry

**DOI:** 10.1038/s41598-022-13999-0

**Published:** 2022-06-15

**Authors:** Yael Lustig-Barzelay, Ifat Sher, Inbal Sharvit-Ginon, Yael Feldman, Michael Mrejen, Shada Dallasheh, Abigail Livny, Michal Schnaider Beeri, Aron Weller, Ramit Ravona-Springer, Ygal Rotenstreich

**Affiliations:** 1 Goldschleger Eye Institute, Sheba Medical Center, 52621 Tel-Hashomer, Israel; 2 Sackler Faculty of Medicine, Tel Aviv University, Tel Aviv, Israel; 3 The Nehemia Rubin Excellence in Biomedical Research, TELEM Program, Sheba Medical Center, Tel Hashomer, Israel; 4 The Joseph Sagol Neuroscience Center, Sheba Medical Center, Tel Hashomer, Israel; 5 Department of Psychology, Bar-Ilan University, Ramat Gan, Israel; 6 Condensed Matter Physics Department, School of Physics and Astronomy, Tel-Aviv University, Tel Aviv, Israel; 7 Department of Diagnostic Imaging, Sheba Medical Center, Tel Hashomer, Israel; 8 Sagol School of Neuroscience, Tel Aviv University, Tel Aviv, Israel; 9 Department of Psychiatry, The Icahn School of Medicine at Mount Sinai, New York, USA; 10 Gonda Brain Research Center, Bar Ilan University, Ramat Gan, Israel; 11 Memory Clinic, Sheba Medical Center, Tel Hashomer, Israel

**Keywords:** Alzheimer's disease, Predictive markers

## Abstract

Currently there are no reliable biomarkers for early detection of Alzheimer's disease (AD) at the preclinical stage. This study assessed the pupil light reflex (PLR) for focal red and blue light stimuli in central and peripheral retina in 125 cognitively normal middle age subjects (45–71 years old) at high risk for AD due to a family history of the disease (FH+), and 61 age-similar subjects with no family history of AD (FH−) using Chromatic Pupilloperimetry coupled with Machine Learning (ML). All subjects had normal ophthalmic assessment, and normal retinal and optic nerve thickness by optical coherence tomography. No significant differences were observed between groups in cognitive function and volumetric brain MRI. Chromatic pupilloperimetry-based ML models were highly discriminative in differentiating subjects with and without AD family history, using transient PLR for focal red (primarily cone-mediated), and dim blue (primarily rod-mediated) light stimuli. Features associated with transient pupil response latency (PRL) achieved Area Under the Curve Receiver Operating Characteristic (AUC-ROC) of 0.90 ± 0.051 (left-eye) and 0.87 ± 0.048 (right-eye). Parameters associated with the contraction arm of the rod and cone-mediated PLR were more discriminative compared to parameters associated with the relaxation arm and melanopsin-mediated PLR. Significantly shorter PRL for dim blue light was measured in the FH+ group in two test targets in the temporal visual field in right eye that had highest relative weight in the ML algorithm (mean ± standard error, SE 0.449 s ± 0.007 s vs. 0.478 s ± 0.010 s, p = 0.038). Taken together our study suggests that subtle focal changes in pupil contraction latency may be detected in subjects at high risk to develop AD, decades before the onset of AD clinical symptoms. The dendrites of melanopsin containing retinal ganglion cells may be affected very early at the preclinical stages of AD.

## Introduction

Over 35 million people world-wide live with Alzheimer's Disease (AD), the most common form of dementia^1^. Treatments have limited effects on symptoms, and while the FDA recently approved a drug that targets successfully the pathology of AD^2^, there is no sufficient evidence supporting cognitive benefits. The lack of cognitive benefits may be attributed, among other factors, to the introduction of treatment at the clinical phases of the disease, when the brain is overwhelmed by pathology, which begins decades before the clinical symptoms become explicit. Early detection of AD, at the pre-clinical stage, or identification of patients at risk, is critical for implementing preventative measurements, and for effective clinical trials of disease modifying treatments^3,4^. First degree family history of AD was found to be a major risk factor for developing the disease^5–9^. However, currently there are no clinical tests that can predict AD development.

The retina is considered an accessible extension of the central nerve system. As such, previous studies investigated whether retinal and optic nerve structural changes can be identified at pre-clinical stages of AD, with results remaining inconclusive^10–12^. The Pupillary Light Reflex (PLR) is mediated by intrinsically photosensitive retinal ganglion cells (ipRGCs) that receive internal inputs from intrinsic activation of melanopsin, and external inputs from the outer retinal photoreceptors, namely the rods and the cones. The ipRGCs mediate the PLR and the light entrainment of the circadian rhythm. Recent studies demonstrated abnormal circadian function and loss of ipRGCs in post-mortem eyes of AD patients^13^. Pupillometry is a simple, fast, and non-invasive test that is gaining interest as a potential readily accessible tool for assessment of neurodegenerative processes in the retina and the brain^14,15^. Several studies indicated changes in the PLR in response to white light stimuli in AD patients, yet the results remain inconclusive, most likely due to differences in methodologies (reviewed in Ref.^15^). Thus, some studies indicated reduced baseline pupil diameter in AD patients compared with controls^16–18^, whereas others reported insignificant differences in baseline pupil size^19,20^. Several studies demonstrated increased or decreased contraction latency, decreased contraction amplitude and reduced mean contraction velocity^17,19–24^. Differences in re-dilation phase after light offset were also reported^16,17^ and pupil recovery time was found to be longer in autosomal dominant AD mutation carriers^25^. Finally, two studies reported that Mini Mental State Examination (MMSE) and Wechsler memory scores in AD patients correlated moderately with acceleration, velocity and latency of pupil contraction^19,26^. However, another study that compared between the PLR of pre-clinical AD patients and healthy controls, using flashes of white light, found no differences in the contraction and re-dilation phases^27^.

Chromatic pupillometry enables the assessment of the relative contribution of cones, rods, and melanopsin to the PLR by measuring the PLR for red, dim and bright blue light stimuli, respectively^28–32^. Pupillometry studies using full-field (> 90°) blue and red-light stimuli reported contradictory results. Thus, Oh et al. reported no difference between the PLR recorded in 10 subjects over 60 years old at high risk to develop AD, based on abnormal values of cerebrospinal fluid (CSF) amyloid β_1–42_ and total Tau protein^33^. By contrast, Romagnoli et al. reported attenuated rod mediated PLR, in 26 mild-moderate AD patients (mean age 69.3 years old) compared to age similar control^34^. Since large (> 90°) light stimuli were used in those studies, the PLR recorded reflected the mean response of ipRGCs across the retina, masking possible focal changes in intrinsic and extrinsic activation of ipRGCs^32,35^.

Chromatic pupilloperimetry measures the PLR for 54 small (0.43°) dim and bright red and blue light stimuli presented at a 30° visual field, thereby allowing examination of the rod-, cone- and melanopsin mediated-PLR at various retinal locations. Previous studies indicated the feasibility of detecting focal changes in retinal function in patients with retinal and macular degeneration by utilizing chromatic pupilloperimetry with high sensitivity and specificity^35–38^. Those studies indicated that the transient PLR obtained in response to short (1 s) focal low intensity (dim) blue light stimuli is mainly mediated by rod photoreceptors, and cone photoreceptors significantly contribute to the PLR for focal red light stimuli^35–39^. Melanopsin-contribution to the PLR can be assessed by presenting bright blue light stimuli^32^. In this study, we combined the use of chromatic pupilloperimetry, with machine learning-based analysis, and demonstrated a potential novel approach for identifying the existence of a high risk for developing AD, in a large cohort (n = 186) of cognitively asymptomatic middle-aged subjects based on the PLR for focal chromatic light stimuli.

## Results

### Demographic, cognitive features and ophthalmic analysis

One hundred and eighty-six Israel Registry for Alzheimer’s Prevention (IRAP) participants [mean age 59.7 (SD 6.37) and 64.5% (n = 120) women] were included in the analysis. Of these, 125 (67.2%) had a family history of AD (family history positive, FH+), and 61 (32.8%) had no family history of AD (family history negative, FH−). Participant's age span was 44–71 years old (YO). There was no significant difference in age between groups (Table 1). However, FH− participants had more years of education (p = 0.0216). The proportion of women in the IRAP study was higher than men, especially in the FH− group. No significant differences in total gray matter and hippocampal volumes were observed between groups after adjustment for total intracranial volume (Table 1).

**Table 1:** Descriptive characteristics of the study cohort by Alzheimer disease (AD) family history.

	FH+^a^	FH−^a^	p-value
**Demographic characteristics**			
N	125	61	
Age mean (SD)^b^	59.93 (± 6.69)	59.08 (± 5.56)	0.385
Female participants	75 (60%)	45 (74%)	0.065
Education years	15.84 (± 3.30)	17.03 (± 3.26)	**0.022**
**Brain MRI-volumetric**			
N	97	31	
Age mean (SD^b^)	59.23 (± 6.79)	58.32 (± 6.24)	0.495
Female participants	56 (58%)	21 (68%)	0.401
Gray matter^c^, mean (SD)	587.8 (53.39)	568.8 (51.3)	0.317
Hippocampus^c^, mean (SD)	0.322 (0.032)	0.317 (0.033)	0.792

^a^FH = Family history of AD, ^b^Age = age at pupilloperimetry testing, ^b^SD = standard deviation; ^c^mean of total volumes in cubic centimeters.

In an unadjusted analysis, FH+ cognitive performance was comparable to the performance FH− in the three cognitive domains. No significant differences in cognitive scores were observed between groups after adjustment for age, sex, and education (Supplementary Table 1). In addition, no significant differences in best corrected visual acuity (BCVA), refraction, intraocular pressure (IOP) or Humphrey visual field mean deviation (MD) scores were observed between groups, with and without adjustment for age, sex, and education (Supplementary Table 2).

### Machine learning models

**Figure 1:**
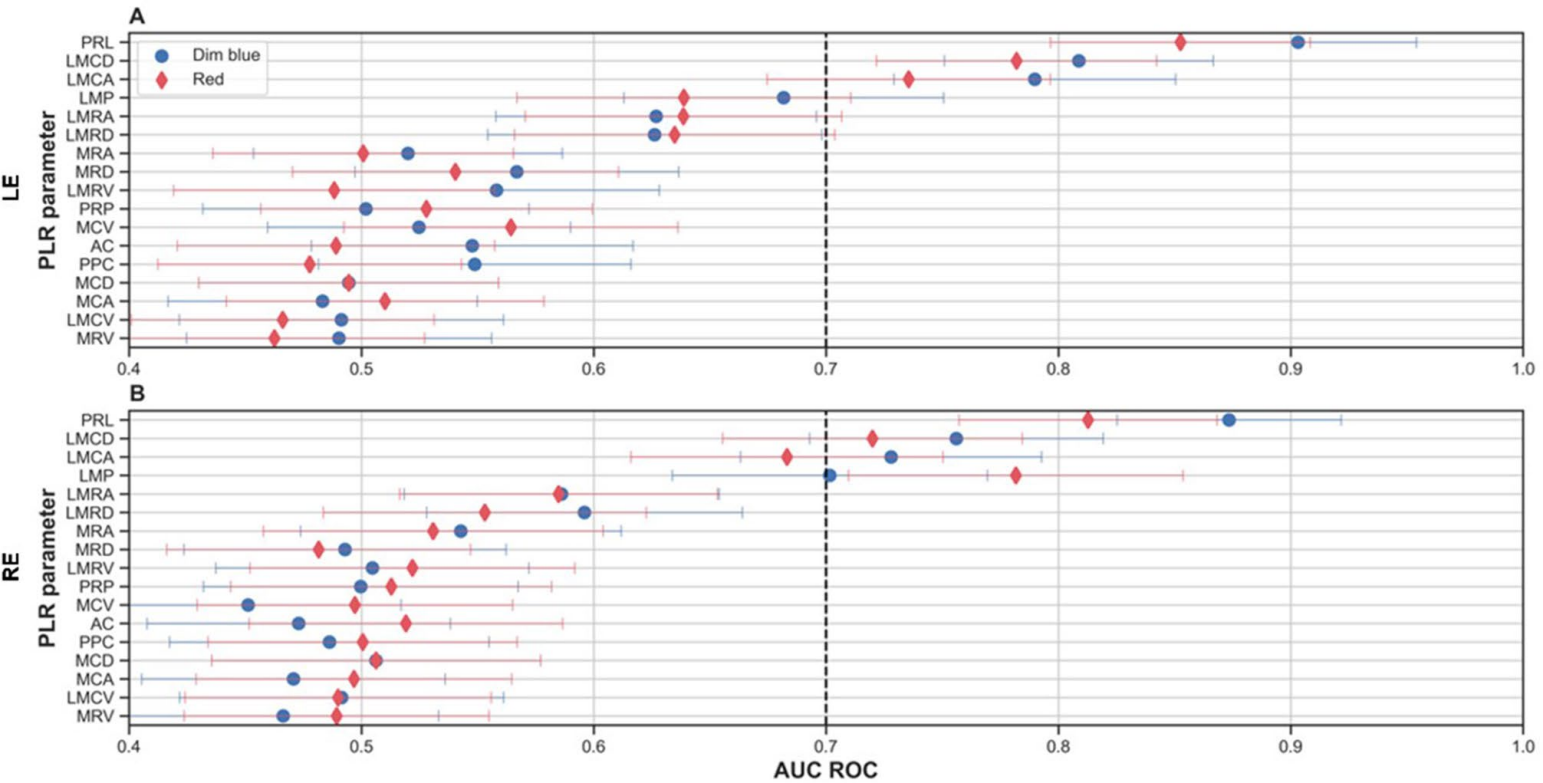
The mean Area Under the Receiver's Operating Curve (AUC-ROC) for each PLR parameter for dim red (red diamond) and blue (blue circle) light stimuli in the left (**A**) and right (**B**) eyes was measured using the bootstrapping process (depicted in Fig. 6). Error bars represent the Standard Deviation (SD) for each parameter. The black dashed lines highlight the cutoff of AUC-ROC = 0.7.

The Area Under the Curve Receiver Operating Characteristic (AUC-ROC) curve for each one of our 136 trained models is presented in Table 2 and Supplementary Table 3. The models that achieved the highest discriminative performances were the latency of the transient pupil contraction from light onset (Pupil Response Latency, PRL) in response to dim blue (B_PRL) and red (R_PRL) light (Fig. 1). The AUC-ROC values of these two parameters were 0.90 ± 0.051, CI of [0.81, 0.98] (left eye), 0.87 ± 0.048, CI of [0.77, 0.95] (right eye) for the blue light, and of 0.85 ± 0.05, CI of [0.75, 0.93] (left eye), 0.81 ± 0.06, CI of [0.68, 0.91] (right eye) in response to red light.

**Table 2:** Performance values of all classification models based on single PLR parameters in response to illumination with dim blue and red light.

PLR parameter		Left eye		Right eye	
		AUC-ROC 95% CI	AUC-ROC Mean ± SD	AUC-ROC 95% CI	AUC-ROC Mean ± SD
Dim blue	AC	[0.41, 0.68]	0.548 ± 0.069	[0.34, 0.60]	0.473 ± 0.065
	LMCA	[0.66, 0.90]	0.790 ± 0.061	[0.60, 0.85]	0.728 ± 0.065
	LMCD	[0.68, 0.91]	0.809 ± 0.058	[0.63, 0.87]	0.756 ± 0.063
	LMCV	[0.35, 0.62]	0.491 ± 0.070	[0.35, 0.63]	0.491 ± 0.070
	LMP	[0.54, 0.81]	0.682 ± 0.069	[0.55, 0.82]	0.702 ± 0.068
	LMRA	[0.49, 0.76]	0.627 ± 0.069	[0.45, 0.71]	0.586 ± 0.068
	LMRD	[0.48, 0.76]	0.626 ± 0.072	[0.46, 0.72]	0.596 ± 0.068
	LMRV	[0.41, 0.69]	0.558 ± 0.070	[0.37, 0.63]	0.505 ± 0.067
	MCA	[0.35, 0.61]	0.483 ± 0.067	[0.34, 0.60]	0.471 ± 0.065
	MCD	[0.37, 0.62]	0.495 ± 0.065	[0.36, 0.63]	0.506 ± 0.071
	MCV	[0.39, 0.65]	0.525 ± 0.065	[0.32, 0.58]	0.451 ± 0.066
	MRA	[0.39, 0.65]	0.520 ± 0.066	[0.40, 0.68]	0.543 ± 0.069
	MRD	[0.42, 0.70]	0.567 ± 0.070	[0.36, 0.63]	0.493 ± 0.069
	MRV	[0.36, 0.62]	0.490 ± 0.066	[0.33, 0.59]	0.466 ± 0.067
	PPC	[0.41, 0.68]	0.549 ± 0.067	[0.35, 0.62]	0.486 ± 0.069
	PRL	[0.79, 0.98]	0.903 ± 0.051	[0.77, 0.95]	0.873 ± 0.048
	PRP	[0.36, 0.64]	0.502 ± 0.070	[0.36, 0.63]	0.500 ± 0.068
Dim red	AC	[0.36, 0.63]	0.489 ± 0.068	[0.38, 0.65]	0.519 ± 0.067
	LMCA	[0.61, 0.84]	0.735 ± 0.061	[0.54, 0.80]	0.683 ± 0.067
	LMCD	[0.65, 0.89]	0.782 ± 0.060	[0.58, 0.84]	0.720 ± 0.065
	LMCV	[0.34, 0.59]	0.466 ± 0.065	[0.36, 0.62]	0.490 ± 0.066
	LMP	[0.49, 0.77]	0.639 ± 0.072	[0.63, 0.90]	0.782 ± 0.072
	LMRA	[0.49, 0.76]	0.639 ± 0.068	[0.44, 0.71]	0.585 ± 0.068
	LMRD	[0.49, 0.76]	0.635 ± 0.069	[0.41, 0.69]	0.553 ± 0.069
	LMRV	[0.35, 0.62]	0.488 ± 0.069	[0.38, 0.65]	0.522 ± 0.070
	MCA	[0.37, 0.64]	0.510 ± 0.068	[0.36, 0.63]	0.497 ± 0.068
	MCD	[0.39, 0.65]	0.526 ± 0.068	[0.38, 0.65]	0.517 ± 0.066
	MCV	[0.41, 0.69]	0.564 ± 0.072	[0.37, 0.62]	0.497 ± 0.068
	MRA	[0.37, 0.63]	0.501 ± 0.065	[0.39, 0.67]	0.531 ± 0.073
	MRD	[0.40, 0.67]	0.540 ± 0.070	[0.35, 0.61]	0.482 ± 0.065
	MRV	[0.33, 0.59]	0.462 ± 0.065	[0.36, 0.62]	0.489 ± 0.066
	PPC	[0.35, 0.61]	0.478 ± 0.065	[0.37, 0.63]	0.501 ± 0.067
	PRL	[0.73, 0.95]	0.852 ± 0.056	[0.69, 0.90]	0.813 ± 0.056
	PRP	[0.38, 0.66]	0.528 ± 0.071	[0.38, 0.64]	0.513 ± 0.069

Data are presented as the confidence interval (CI), mean ± standard deviation (SD) and AUC-ROC, using 2000 iterations, for each one of 68 trained models. Each row indicates one PLR parameter. Each parameter appears twice, induced by dim blue and red light.

Other models that achieved acceptable discriminative values (AUC-ROC ≥ 0.7) for both eyes were those related to the latency of maximal pupil contraction acceleration (LMCA) for dim blue light (B_LMCA) and red light stimuli ( R_LMCA), and the latency of maximal pupil contraction deceleration (LMCD) for dim blue (B_LMCD) and red (R_LMCD) light stimuli. The latency of maximal pupil contraction (LMP) for dim blue (B_LMP) and red (R_LMP) light stimuli achieved a discriminative AUC-ROC in the dataset based on right eye measurements (mean ± SD 0.702 ± 0.068 and non-discriminative AUC-ROC based on left eye measurements (0.682 ± 0.069). The remaining parameters related to the latency of pupil contraction for dim blue and red light, namely the latency of maximal contraction velocity (LMCV) for dim blue (B_LMCV) and red (R_LMCV) light stimuli, gave AUC-ROC < 0.7 (Fig. 1). All models derived from PLR for bright red or blue light stimuli gave very low AUC-ROCs (≤ 0.45) resembling random classification (Supplementary Table 3).

### Focal analysis

**Figure 2:**
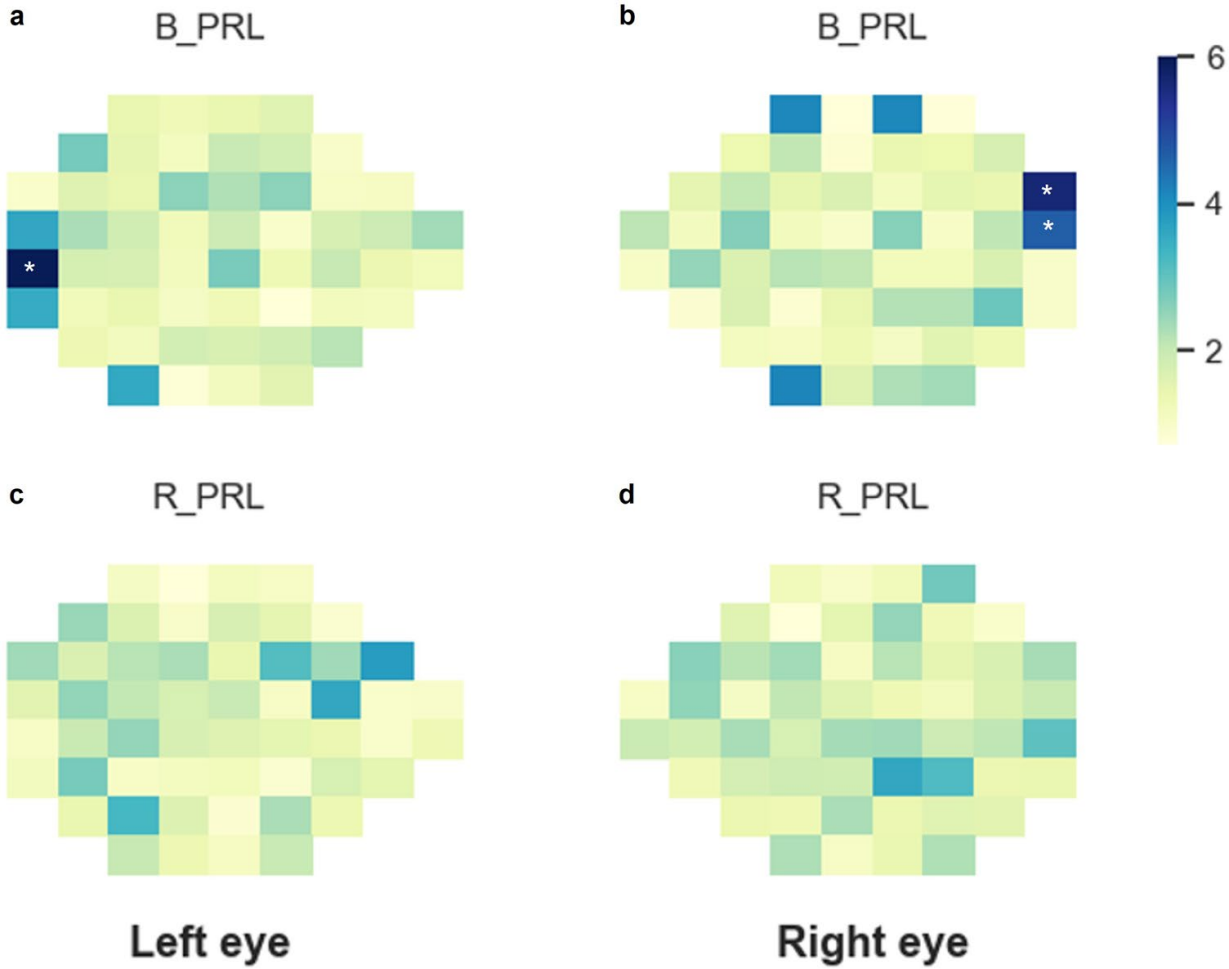
The relative weight given by the machine-learning model to each one of the 54 retinal test-targets for Pupil Response Latency (PRL) in the left (**a**,**c**) and right eye (**b**,**d**) for dim blue (**a**,**b**, B_PRL) and dim red light stimuli (**c**,**d**, R_PRL). The weights were averaged over the 2000 trials that were used to produce the 95% CI, as depicted in Fig. 6. Color-coding indicates higher weights in darker colors. White asterisks indicate the targets with highest weight for the dim blue light (**a**,**b**).

Figure 2 presents the two most prominent parameters, namely the pupil response latency (PRL) for dim blue (B_PRL, Fig. 2a,b) and red (R_PRL, Fig. 2c,d) light stimuli and the weights given by the machine-learning model to each one of the 54 test-targets measured.

Overall, the test targets most discriminative of a positive AD family history in response to dim blue light were located in the periphery of the 24-degree visual field, particularly in the temporal side (nasal side of the retina). In the right eye, the mean PRL for dim blue light in the two most discriminative visual field targets (weight > 0.47) in the temporal VF (marked with dark blue shade and asterisks in Fig. 2b) was significantly shorter in the FH+ group compared to the FH- group (mean ± standard error, SE 0.449 s ± 0.007 s vs. 0.478 s ± 0.01 s, p = 0.038, Table 3). In the left eye a trend to shorter mean PRL was recorded in the most discriminative visual field target in the FH+ group compared to the FH− group, but the difference did not reach statistical significance (mean ± standard error, SE 0.427 s ± 0.013 s vs. 0.457 ± 0.019 s, p = 0.168, Table 3). Similar results were obtained in both eyes with and without adjustment for age (Table 3).

**Table 3:** Difference in Pupil Response Latency (PRL) between groups at targets with highest weights given by the machine-learning algorithm.

Model	Eye	Group	Mean ± SE	95% CI	*p*
I: Unadjusted	Right	FH−	0.477 ± 0.009	0.459–0.495	0.038*
		FH+	0.450 ± 0.007	0.435–0.464	
	Left	FH−	0.456 ± 0.014	0.424–0.489	0.168
		FH+	0.427 ± 0.016	0.400–0.454	
II: Adjusted for age	Right	FH−	0.478 ± 0.010	0.458–0.498	0.038*
		FH+	0.449 ± 0.007	0.436–0.463	
	Left	FH−	0.457 ± 0.019	0.420–0.494	0.197
		FH+	0.427 ± 0.013	0.402–0.457	

Data are presented as the mean ± standard error (SE), and 95% confidence interval (CI) of the pupil response latency (PRL) in seconds recorded in each group in the right eye in the two discriminative visual field targets and the left eye in the discriminative visual field target (see Fig. 2). *p < 0.05 following Bonferroni correction.

In response to dim red light stimuli, discriminative test targets were detected throughout the retina with no specific pattern (Fig. 2c,d).

## Discussion

Using chromatic pupilloperimetry testing in conjunction with machine learning algorithms we demonstrated that multi-parametric analysis of PLR kinetics for focal red and blue light stimuli can differentiate with high discriminative power cognitively asymptomatic individuals at high risk for AD due to a parental family history from individuals with no family history of AD.

The main contributions of this study are three; first, our prediction model could classify normally cognitively asymptomatic individuals according to the existence of AD family history, which is a major risk factor for developing the disease. Second, the prediction model achieving highest AUC-ROC was based on parameters pertaining to the latency of the transient PLR contraction phase. Third, the ML identified two test targets in the temporal visual field (nasal retina) in the right eye with significantly shorter pupil response latency in subjects at high risk for AD.

Our findings that the contraction latency parameters were most discriminative between groups are in agreement with published studies demonstrating changes in AD patients compared to healthy controls in PLR parameters related to the afferent arm of the reflex. Among them, some studies reported changes in latency-related PLR parameters^21,22^, while others found changes in PLR parameters pertaining to the contraction phase, but not to the latency of the response^16,17,23^. To the best of our knowledge this is the first study that shows an association between shorter latency of pupil contraction and AD risk due to a parental family history in asymptomatic middle-aged adults. Differences between PLR findings in published papers and our work may be related to the study population (AD risk vs. AD patients) and pupillometry method, i.e. full field white or chromatic light stimuli vs. focal red and blue stimuli that enables to assess rod, cone and melanopsin mediated PLR at central and peripheral retinal locations, as well as the advancement in capturing the pupil response.

The PLR parameter that achieved the highest performance rates by our learning algorithm for both eyes was the latency of the transient PLR, suggesting an effect on the contraction arm of the PLR, which is attributed to parasympathetic system. This finding is supported by studies demonstrating that AD Patients have reduced synthesis of Acetylcholine^40^, the key neurotransmitter in the para-sympathetic nervous system^41^. AD affects the cholinergic system^42^, and specifically the cholinergic Edinger Westphal Nucleus (EWN)^43,44^, which is involved in the control of the pupil contraction^45^. Our findings are in accordance with published studies with patients with probable AD reporting attenuated pupil contraction acceleration^22,24,46^, shorter PLR latency and lower amplitude of pupil contraction^24^.

Using the pupil response latency (PRL) parameter, the highest performance values were obtained for both eyes were in response to dim blue light with lower, yet still very high performance for red light. These results may indicate that changes in the rod PLR pathway may be associated with high risk for AD. Interestingly, the most discriminative test targets of a positive AD family history in response to dim blue light were located in periphery of the visual field, in the temporal area, whereas in response to dim red light stimuli, discriminative test targets were detected across the visual field with no specific pattern. These finding may suggest attenuated function of the neuronal circuits downstream of rods in the nasal retina in subjects at high risk for AD. Furthermore, these findings indicate the possibility of running a shorter chromatic pupilloperimetry test, using a smaller number of test targets spots in the temporal visual field, that may suffice for screening using such classification algorithm. This should be validated in further studies. In accordance with our results, aberrant function of the rod pathway was also observed in full-field electrophysiology studies in mouse models of AD, where abnormal dark adapted retinal function was recorded, whereas cone photoreceptor function and signal transmission to second order neurons was maintained^47,48^. Our data are also in accordance with the recent study of Zhang et al. who reported the deposition of Aβ plaques around photoreceptors in the retina of the APP23 mouse model of AD. The Aβ plaques were detected in the outer retina at the age of 9 months, 3 months before their appearance in the mouse brain. Moreover, immunohistochemistry and ERG analyses indicated a progressive degeneration of rods that preceded the degeneration of cones and bipolar cells in these mice^49^. The reasons for the more significant effect of AD risk on rods than cones, and specifically on the more peripheral rods remains unknown. Several mechanism were suggested for the attenuated rod mediated PLR in AD^34^. The rod-mediated PLR may depend more on the distal dendrites and may thus be more sensitive to dendritic degeneration, and perhaps more so in the peripheral retina where the density of iPRGCs is lower and their dendritic fields are larger^50^. Another possible explanation could be that rod-bipolar cells are more susceptible to AD pathology than the cone-bipolar cells.

While almost all models based on PLR parameters gave either discriminative values or non-discriminative values for both eyes, the LMP (Latency of Maximal Pupil response) parameter was found to be discriminative in the dataset based on right eye measurements (AUC-ROC = 0.702) and non-discriminative based on left eye measurements (AUC-ROC = 0.682). While these values are relatively close to each other, the threshold of 0.7 that was set to differentiate between models that achieved discriminative values suggested that the model for the left eye is non-discriminative while the model of the right eye is^51^. Since a binary threshold was set, it is reasonable that for certain parameters we would get results of this form.

Since the PLR for bright red and blue light had low discrimination between groups, our data suggest that, the extrinsic activation of ipRGCs is affected in people at high risk to develop AD, whereas the melanopsin-mediated intrinsic activation of ipRGCs remains intact. Our findings are in accordance with the finding of Romagnoli et al. who reported normal melanopsin-mediated PLR and aberrant rod-mediated PLR in mild-moderate AD patients using full-field chromatic pupillometry^34^. This may suggest that defects in ipRGC dendrites may be an early event in AD pathology, decades before cognitive decline. Interestingly, RGC dendritic atrophy preceding RGC cell loss was observed in three mouse models of AD, at early stages of the disease, in parallel with hippocampal dendritic spine loss^52,53^.

All parameters with AUC-ROC > 0.7 were related to PLR latency suggesting a possible attenuation of the speed of neuronal signaling associated with high risk of AD disease. The only latency-related parameter that had AUC-ROC < 0.7 for both eyes was the latency of maximal velocity (LMCV). Several studies indicated that the LMCV parameter differentiates with high specificity and sensitivity between healthy controls and patients with photoreceptor degeneration^36,38^. SD-OCT imaging for all participants included in the current study did not indicate any sign of photoreceptor degeneration. Hence, our findings that the LMCV features did not differentiate well between the study groups, suggest that the PLR pathology at the subjects at high risk of AD may occur downstream to the photoreceptors, at the bipolar/amacrine or ipRGCs dendrites. These findings are in accordance with the findings of McAnany et al., who have most recently reported post-receptor abnormalities in retinal function in a mouse model of AD under dark adaptation using full-field ERG^47^.

Our study has several limitations. First, our cohort included 186 participants. Since each participant had PLR examinations done on both eyes, we ran our experiments on two datasets, a dataset for the left eye and for the right eye, with each dataset consisting of data extracted from 186 eyes. Although our data set was rather large, verification of the results in larger cohorts may assist in strengthening our conclusions. In addition, our study was cross sectional. Hence, we can only speculate that pupil function alterations predict the development of AD, but this must be tested in a longitudinal design, which is ongoing in the IRAP study^54^. Another limitation of our study is that it included only Caucasian subjects, which are the majority in the Israeli population. Hence, future studies including non-Caucasian individuals are required to expand the generalizability of our findings.

A third limitation concerns the ratio between subjects with and without AD family history. Most participants in our study had a confirmed AD family history, and they accounted for 67.2% of the study's participants. This number does not reflect the normal proportion of this condition in the population. This is attributed to the fact that our study participants are part of the IRAP cohort, which was formed as part of a longitudinal study^54^. Participants are required to undergo a battery of clinical testing including cognitive assessment, MRI and blood tests every several years. In this type of study design recruiting healthy subjects in more challenging.

To the best of our knowledge, this is the first attempt to identify focal changes in the rod-cone and melanopsin-mediated PLR in cognitively normal, asymptomatic participants at high risk for AD. Using chromatic pupilloperimetry testing in a relatively large cohort and artificial intelligence tools, our study suggests that individuals at high risk to develop AD have specific changes, already in midlife, in the latency of PLR for focal dim red and blue light stimuli. Rod-circuits are specifically affected at the nasal retina whereas the cone-circuits are affected across the central retina, and melanopsin function remains unaffected at this early stage.

## Methods

### Study design and precipitants

#### Participants

The study was approved by the Sheba Medical Center and MHS institutional review board (IRB) committees, and all participants signed an informed consent. The described research adhered to the tenets of the Declaration of Helsinki. Participants (N = 301) are from the IRAP study, and all are members of the Maccabi Healthcare Services (MHS), the second largest HMO in Israel^54^. Briefly, participants were recruited through the MHS homepage and word of mouth. They were either offspring of AD patients (family history positive, FH+) or subjects with no parental history of AD (family history negative, FH−). Potential participants underwent questioning regarding their age, MHS membership, and parental dementia. Medical records of their parents were provided to the study team and a dementia questionnaire (DQ) was administered to determine the likely presence of AD in parents of potential study volunteers. The DQ is a validated, informant-based instrument^55^. The medical history, diagnostic workup and the DQ were reviewed by the study team to reach a probable AD diagnosis (according to NINCDS-ADRDA criteria) or lack of, in parents of potential participants. In case of partial information about dementia type or with dementia other than AD, the potential participant was excluded from the study. Also excluded were offspring of parents with an AD onset before the age of 55 (which are assumed to have familial early onset AD). Siblings were excluded from the study for avoiding confounding results (the first eligible offspring who volunteered was included in the study). Detailed methods are provided in Ref.^54^.

##### Brain MRI

Brain MRI was performed as part of the IRAP parent study. One hundred and twenty-seven participants had MRI done within the same year of chromatic pupilloperimetry testing and their data was included in the study. MRI scans were acquired on a three Tesla whole body MRI system (GE Signa HDxt, version 16 VO2) equipped with an eight-channel radio frequency head coil. The MRI scanning included T1-weighted imaging and T2-weighted-fluid-attenuated inversion recovery (T2-FLAIR) for clinical purposes^39^. The 3D T1-weighted images underwent the common processing pipeline for voxel-based morphometric analysis^56^, using CAT12^57^ toolbox implemented in SPM12 software (https://www.fil.ion.ucl.ac.uk/spm/software/spm12/) in the MATLAB environment (The MathWorks, Inc., Natick, Massachusetts, United States). Gray-matter, white-matter, and cerebrospinal fluid components were obtained to calculate the total intracranial volume in the native space. Gray matter and Hippocampal volumes were extracted from the smoothed modulated and normalized images, and were entered into group comparisons adjusting for total intracranial volume.

##### Cognitive assessment

Subjects underwent cognitive assessment on the same day as chromatic pupilloperimetry testing. The neuropsychological tests included: Rey Auditory Verbal Learning test—immediate and delayed recall and recognition^58^, Trail making tests A and B, the digit symbol substitution test^59^ and the digit span tests forwards and backwards^60.^ The tests were summarized into executive functions (EF), working memory (WM), and episodic memory (EM) domains. The scores of the neuropsychological tests were transformed into Z scores and averaged for each domain, as described in Ref.^54^.

##### Ophthalmologic examination

Participants underwent a complete ophthalmologic examination on the same day as chromatic pupilloperimetry testing to exclude ocular pathologies including assessment of best-corrected visual acuity (BCVA), color vision (Farnsworth D15 test), pupillary reflexes, intraocular pressure (Goldmann applanation tonometry), Humphrey perimetry (24-2 Swedish interactive threshold algorithm test, SITA standard protocol, Humphrey Field Analyzer II, Swedish interactive threshold algorithm 24-2; Carl Zeiss Meditec, Inc., Jena, Germany) and slit lamp bio-microscopy of the anterior and posterior segment, fundus examination and optical coherence tomography (OCT). Eyes with BCVA of 20/50 or lower, or any ocular pathology were excluded.

**Figure 3:**
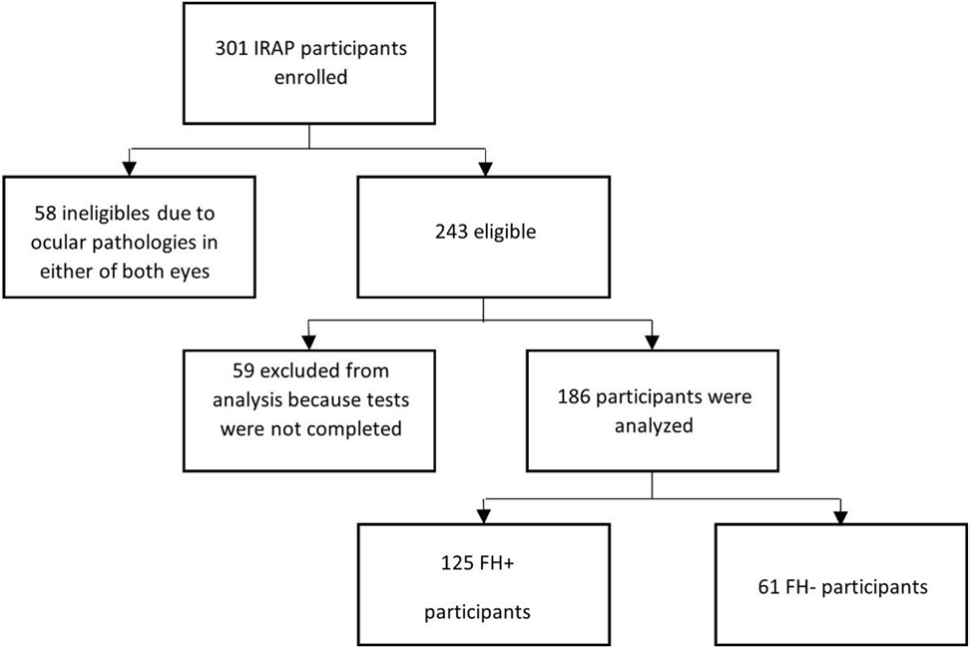
Flow chart of the study recruitment.

Only participants for whom both eyes were eligible for the study were included, a total of 186 patients and 372 eyes (see Fig. 3). Sixty-one of them were FH- and 125 were FH+.

### Chromatic pupilloperimetry

On the same day of cognitive and ophthalmic assessments, each participant underwent chromatic pupilloperimetry testing in both eyes, resulting in a sum of 372 eyes (250 eyes were FH+ and 122 were FH−).

Chromatic pupilloperimetry testing was performed as previously described^35–38^. Briefly, the device is comprised of a Ganzfeld dome apparatus with 76 LEDs (2 mm^2^ diameter), an infrared camera and a fixation light. Each eye was tested separately, and non-tested eye was occluded to prevent PLR due to light perception in the contralateral eye. The test was performed under mesopic conditions using a background light of 0.04 cd/m^2^.

**Figure 4:**
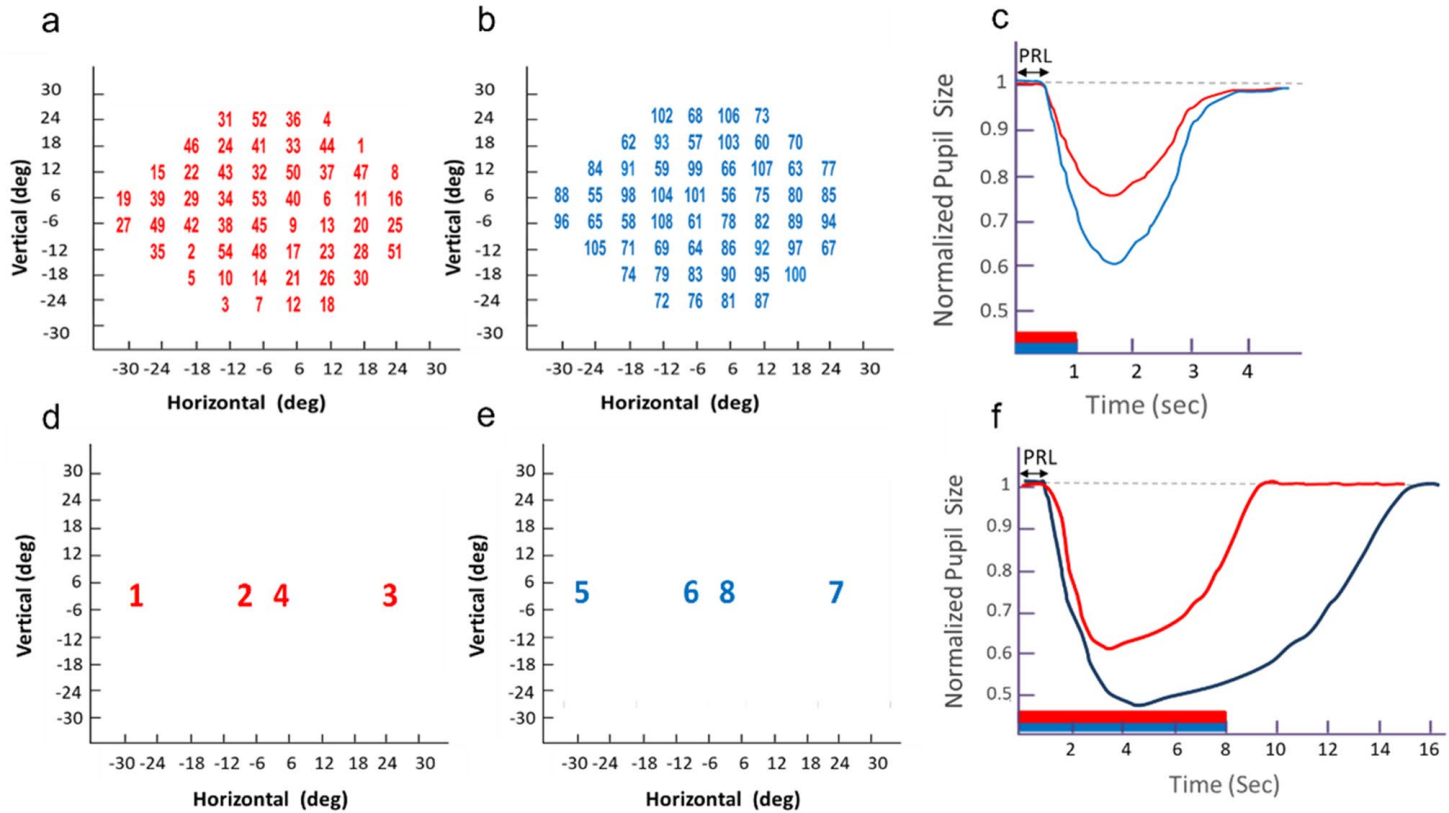
Chromatic pupilloperimetry light stimulus presentation and PLR waveform. The chromatic pupilloperimetry test included 54 test locations in a pattern resembling the Humphrey perimetry 24-2 visual field test. First, the red-light stimuli were presented for 1 s at the indicated sequence (1–54, **a**). Next, dim blue light stimuli were presented for 1 s (55–108, **b**). Finally, bright red-light stimuli were presented for 8 s at two central and two peripheral (**d**) retinal location followed by bright blue stimuli at the same sequence (**e**). The right eye test pattern is presented in panels (**a**,**b**) and (**d**,**e**). (**e**–**c**,**f**–**a**) Schematic presentation of transient (**c**) and sustained (**f**) PLR wave forms obtained in response to dim (**c**) and bright (**f**) red and blue light stimuli. Demonstration of the Pupil Response Latency (PRL) is indicated with arrows (**c**,**f**).

Cone- and rod-mediated PLR were recorded using small (0.43°, Goldmann size III) red (624 nm ± 5 nm, 1000 cd/m^2^) and dim blue (485 nm ± 5 nm, 170 cd/m^2^) light stimuli, respectively. We chose to use 485 nm for testing the rod-mediated PLR to minimize the contribution of green cones^61^. Stimulus intensity was selected based on previous studies indicating that the presentation of red light stimuli at intensity of 1000 cd/m^2^ and blue light stimuli at 170 cd/m^2^ resulted in a substantial percentage of pupil contraction (≥ 10%) in peripheral and central VF locations in healthy subjects^35^. Under these light conditions, cones significantly contributed to the PLR for red light, and the PLR for blue light was mainly mediated by rods with some cone contributions especially at the macula area^35–38^. Light stimuli were presented from individual LEDs, at 54 VF locations selected to match the Humphrey Field Analyzer 24-2 test protocol. Light stimuli were presented for one second, and pupil diameter was captured for four seconds by a computerized infrared high-resolution video camera (Fig. 4a–c, and references^35–38^).

Melanopsin mediated PLR was recorded using bright (6000 cd/m^2^) red (624 nm ± 5 nm) and blue (485 nm ± 5 nm) light stimuli, that were presented at two central and two peripheral retinal locations, for 8 s. The pupil diameter was captured for 16 s (Fig. 4d–f). The blue light stimulus wavelength was chosen based on published studies demonstrating that melanopsin has a peak absorption at 479 nm and its effective in-vivo spectral sensitivity is 487–496 nm depending on subject’s age^62,63^. The PLR for red light at the same intensity was measured as control, since the difference between the sustained PLR for red and blue light stimuli is attributed to melanopsin-mediated response^29,64^.

Test targets in which the subject blinked during the first 2.5 s following light onset were automatically excluded and the targets were automatically retested at the end of the sequence.

Seventeen PLR parameters from both the contraction and dilatation phase were calculated and analyzed for both eyes of each participant. These include pupil contraction and dilation amplitude, velocity, acceleration, and latencies (Supplementary Method 1).

Each one of these parameters was calculated for the PLR response for red, dim and bright blue-light stimuli in each test point resulting in a total of 34 independent parameters for each one of the 54 test point locations for red and dim blue light, and 34 independent parameters for each of the 4 test point locations for bright blue light.

### Machine learning analysis

Using the Chromatic Pupilloperimeter we analyzed the significance of each of the PLR parameters for each of the test point locations, in order to classify participants to either having or not having a family history of AD.

**Figure 5:**
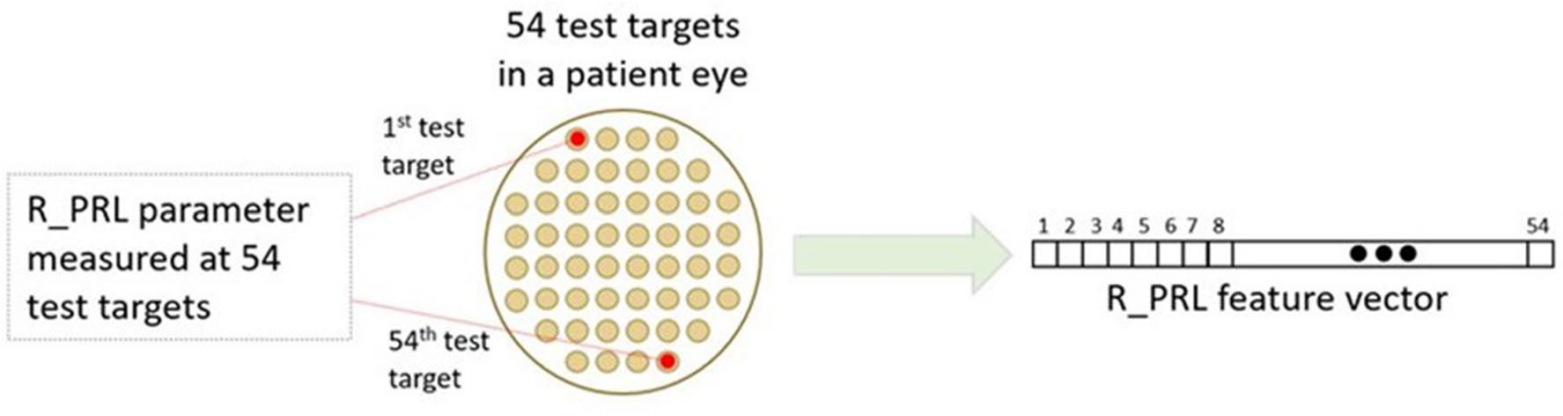
An illustration of a feature vector extraction process from a participant's chromatic pupilloperimetry test. Here a vector for the Pupil Response Latency of a red stimulus (R_PRL), measured in 54 test targets, was generated. The process was repeated per both eyes of each patient.

Each parameter produced either 54 or 4 independent features for the PLR parameter measured at the different test targets of the visual field (VF), as shown in Fig. 5. Our goal was to estimate and compare the predictive accuracy of the different parameters.

We used the AdaBoost algorithm, a widely used boosting algorithm, with a solid theoretical background^65^, for training multiple classifiers. Each classifier received a distinct set of features corresponding to a single PLR parameter. The process was repeated for each eye independently.

For each eye, 17 models were trained based on the 17 PLR parameters extracted using red light, 17 models were trained based on the 17 PLR parameters extracted using dim blue light, 17 models trained based on the 17 PLR parameters extracted using bright blue light, and another 17 models trained based on the 17 PLR parameters extracted using bright red light resulting in a total of 68 × (2 eyes) = 136 different models. We note that a classifier may be trained using a combination of inter parameter features.

Missing data, which were 0.76% from the data, were imputed^66,67^. Imputation was performed using the mean substitution strategy that assigns the overall respondent mean to all missing responses and is the deterministic degenerate form of the linear function with no auxiliary variables. All features were rescaled using min–max normalization to a range of [0, 1].

Discrimination performance of the classification model was measured by the AUC-ROC.

### Statistical analysis

Statistical analysis was done with SPSS v. 20 (IBM, USA). Comparisons between FH+ and FH− groups were performed using chi-square tests for sex category and using a t-test for years of education. General linear model was used to compare MRI gray matter and hippocampal volume adjusting for total intracranial volume (Table 1). For comparisons of the cognitive domains between FH+ and FH− groups we used MANOVA. We then adjusted for age, sex, and education using ANCOVAs. Raw scores of each cognitive test are presented in Supplementary Table 1.

**Figure 6:**
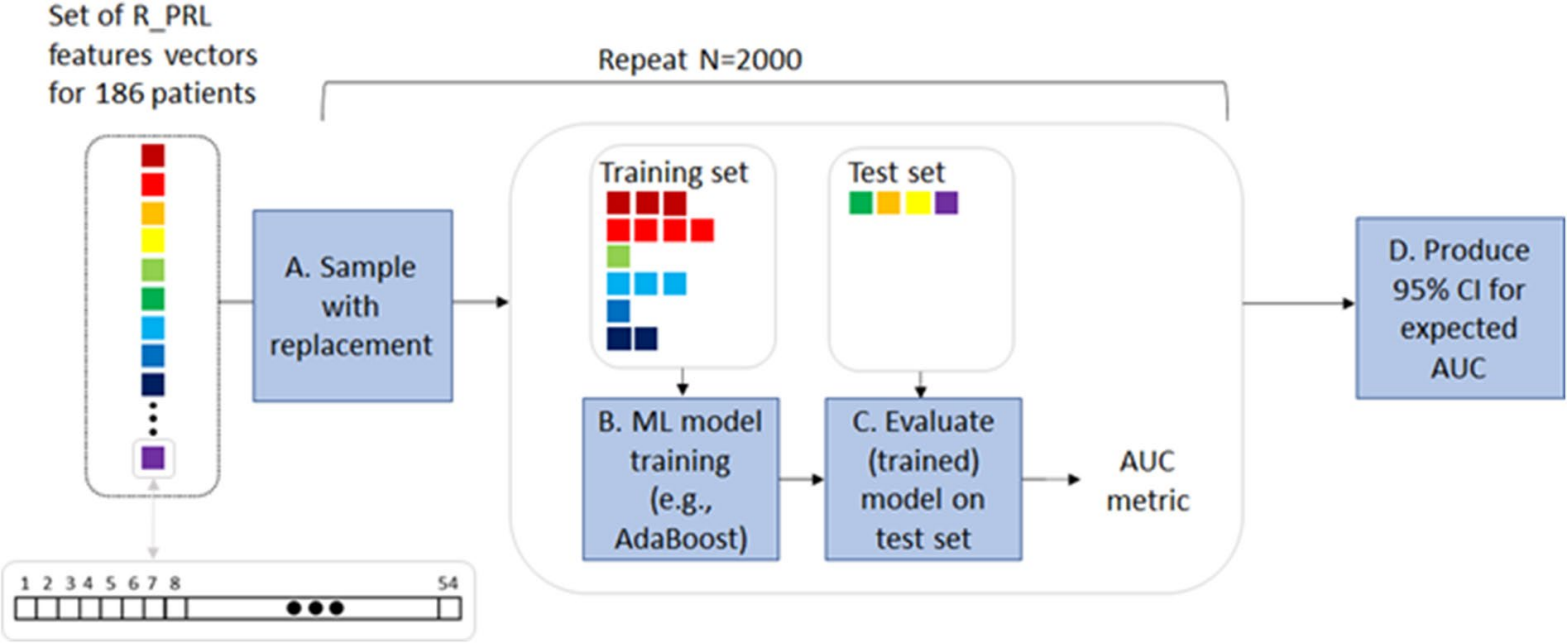
An illustration of producing a confidence interval (CI) for a single PLR parameter (in this case, R_PRL). Given the full set of samples, a training set was created by sampling with replacement, whereas the test set was comprised of all the samples that were not picked in the training set. The model was evaluated on the test set (average size of 88 distinct samples) using the AUC-ROC metric. The process was repeated N = 2000 times to infer a 95% CI. *R_PRL* Pupil Reflex Latency induced by red light, *CI* confidence interval, *AUC-ROC* Area Under the Receiver's Operating Curve.

To assess the confidence interval for the AUC-ROC, we used non-parametric resampling^68^ The general algorithm for a non-parametric bootstrap, applied here, was comprised of the following: (1) Sample *n* observations randomly with replacement from the 186 samples (*n* = 186) to obtain a bootstrap dataset. Sampling was performed in a stratified fashion. (2) Train a classifier using the bootstrap version of the data, test it on the observations that are not part of the bootstrap dataset, to conclude an AUC-ROC measure. (3) Repeat steps (1) and (2) on 2000 replicates, as recommended when calculating CI using bootstrapping^69^, to obtain an estimate of the confidence interval (the entire process is described in Fig. 6). T-test was used to compare the PRL in the discriminative targets and General linear model was used to adjust for age. Bonferroni correction was used to correct for comparing two discriminative test targets in the right eye (Table 3).

## Supplementary Information

Supplementary Information.

## Data Availability

The data that support the findings of this study are available upon a reasonable request from the corresponding author (YR). The data are not publicly available due to them containing information that could compromise research participant privacy.
