# Supplementary material for: Machine learning for comprehensive prediction of high risk for Alzheimer’s disease based on chromatic pupilloperimetry
Source: Sci Rep. 2022 Jun 15;12:9945. doi: 10.1038/s41598-022-13999-0 (PMC9200977; doi:10.1038/s41598-022-13999-0)
Supplement: Supplementary file 1 — Supplementary Information. [file 41598_2022_13999_MOESM1_ESM.docx]

**Machine learning for comprehensive prediction of high risk for Alzheimer’s Disease based on chromatic pupilloperimetry**

Yael Lustig-Barzelay, (1, 2), Ifat Sher (1, 2), Inbal Sharvit-Ginon (3,4), Yael Feldman (1,2), Michael Mrejen (5), Abigail Livny (2,3,6,7), Michal Schnaider-Beeri (3,8), Aron Weller (4,9), Ramit Ravona-Springer (2,3,10), Ygal Rotenstreich (1,2,7)

**Supplementary**

**Supplementary Method 1:**

1. Percentage of Maximal Pupil Contraction (PPC) in %, calculated as:

$$PPC=\frac{Initial Pupil Diameter-Minimum Pupil Diamater}{Initial Pupil Diameter} x 100$$

2. Latency of Maximal Pupil Response (LMP, measured from the time of light onset to time of minimum pupil diameter, in seconds);

3. Maximal Contraction Velocity (MCV in pixel/second, calculated as detailed in references^33–36^);

4. Latency of MCV (LMCV, measured from the time of light onset to time of MCV, in seconds in seconds, as detailed in references^33–36^);

5. Maximal Contraction Acceleration (MCA, in pixel/second^2^);

6. Latency of MCA (LMCA, measured from the time of light onset to time of MCA, in seconds);

7. Maximal Contraction Deceleration (MCD, in pixel/second^2^);

8. Latency of MCD (LMCD, measured from the time of light onset to time of MCD, in seconds);

9. Maximal Relaxation Acceleration (MRA, in pixel/second^2^);

10. Latency of MRA (LMRA, in seconds);

11. Maximal Relaxation Velocity (MRV, in pixel/second);

12. Latency of MRV (LMRV, measured from the time of light onset to time of MRV, in seconds);

13. Maximal Relaxation Deceleration (MRD, in pixel/second^2^);

14. Latency of Maximal Relaxation Deceleration (LMRD, measured from the time of light onset to time of MRD, in seconds);

15. Pupil Response Latency (PRL, measured from the time of light onset to time of onset of pupil contraction, in seconds);

16. Percentage of Pupil Recovery (PPR) was measured at 3.7 seconds following light onset, in % using the formula:

$$PPR=\frac{Pupil Diameter at 3.7sec post light onset x100}{Initial Pupil Diameter}$$

17. Area of the Curve of the PLR waveform (AC);

***Supplementary Table 1: Cognitive factors by AD family history (z scores).***

| Model | Cognitive factor | FH^+^ (n=125) | FH^-^ (n=61) | F | *P* |
| --- | --- | --- | --- | --- | --- |
| I: Unadjusted | EM | 0.032±0.067 | 0.145±0.096 | 0.942 | 0.333 |
|  | WM | -0.008±0.079 | 0.171±0.113 | 1.686 | 0.196 |
|  | EF | 0.064±0.064 | 0.055±0.092 | 0.007 | 0.936 |
| II: Adjusted for age, sex, and education | EM | 0.059±0.063 | 0.088±0.091 | 0.68 | 0.795 |
|  | WM | -0.003±0.078 | 0.159±0.113 | 1.353 | 0.246 |
|  | EF | 0.08±0.06 | 0.021±0.087 | 0.308 | 0.579 |

*Data are resented as the Mean ± Standard Error (SE) for each one of the groups,* F*, and p-value. EM (Episodic Memory), WM (Working Memory), EF (Executive functions).*

***Supplementary Table 2: Ophthalmic testing parameters by AD family history.***

| Model | Ophthalmic Parameter | FH^+^ | FH^-^ | *p* |
| --- | --- | --- | --- | --- |
| I: Unadjusted | BCVA^*^ | 0.04 ± 0.01 | 0.03 ± 0.01 | 0.671 |
|  | Refraction^£^ | -0.42 ± 0.25 | -0.72 ± 0.33 | 0.462 |
|  | IOP^€^ | 11.09 ± 0.27 | 11.13 ± 0.37 | 0.928 |
|  | Humphrey MD^ς^ | -0.81 ± 0.15 | -0.50 ± 0.17 | 0.184 |
| II: Adjusted for age, sex, and education | BCVA^*^ | 0.04 ± 0.10 | 0.034 ± 0.15 | 0.873 |
|  | Refraction^£^ | -0.74 ± 0.27 | -0.35 ± 0.40 | 0.417 |
|  | IOP^€^ | 11.06 ± 0.26 | 11.20 ± 0.39 | 0.772 |
|  | Humphrey MD^ς^ | -0.71 ± 0.17 | -0.31 ± 0.25 | 0.193 |

*Data are resented as the Mean ± Standard Error (SE) for each group,* *and p-value. *BCVA – Best Corrected Visual Acuity (in logMAR), ^£^ Refraction (spherical equivalent, in diopters), ^€^IOP – intraocular pressure (in mmHg),* ^ς^ *Humphrey MD-* *Humphrey perimetry (24-2 Swedish interactive threshold algorithm test, SITA standard protocol) mean deviation.*

***Supplementary Table 3: Performance values of all classification models based on single PLR parameters in response to illumination with bright blue and bright red light stimuli.***

|  | | Left Eye | | Right Eye | | |
| --- | --- | --- | --- | --- | --- | --- |
| PLR parameter | | AUC-ROC  95% CI | AUC-ROC:  Mean ± SD | AUC-ROC  95% CI | AUC-ROC:  Mean ± SD |  |
| Bright Blue | PRL | [0.40,0.66] | 0.54 ± 0.068 | [0.42,0.68] | 0.55 ± 0.066 |  |
|  | LMCA | [0.34,0.60] | 0.48 ± 0.066 | [0.30,0.56] | 0.43 ± 0.066 |  |
|  | Max_PC | [0.37,0.63] | 0.50 ± 0.065 | [0.39,0.66] | 0.53 ± 0.069 |  |
|  | MCA | [0.37,0.64] | 0.51 ± 0.068 | [0.33,0.61] | 0.48 ± 0.068 |  |
|  | MCD | [0.34,0.60] | 0.47 ± 0.065 | [0.43,0.70] | 0.57 ± 0.073 |  |
|  | AC | [0.35,0.61] | 0.48 ± 0.066 | [0.39,0.65] | 0.52 ± 0.065 |  |
|  | LMRD | [0.43,0.70] | 0.57 ± 0.068 | [0.33,0.59] | 0.46 ± 0.066 |  |
|  | LMRV | [0.40,0.67] | 0.54 ± 0.069 | [0.43,0.68] | 0.56 ± 0.065 | |
|  | LMP | [0.44,0.72] | 0.58 ± 0.072 | [0.34,0.59] | 0.47 ± 0.065 | |
|  | LMRA | [0.45,0.70] | 0.58 ± 0.065 | [0.38,0.63] | 0.51 ± 0.064 | |
|  | MRA | [0.35,0.61] | 0.48 ± 0.065 | [0.38,0.63] | 0.51 ± 0.062 | |
|  | MCV | [0.32,0.57] | 0.44 ± 0.064 | [0.36,0.63] | 0.50 ± 0.066 | |
|  | MRV | [0.34,0.58] | 0.47 ± 0.064 | [0.36,0.63] | 0.50 ± 0.068 | |
|  | PRP | [0.42,0.69] | 0.56 ± 0.071 | [0.33,0.59] | 0.46 ± 0.065 | |
|  | LMCD | [0.32,0.57] | 0.45 ± 0.064 | [0.32,0.59] | 0.47 ± 0.068 | |
|  | LMCV | [0.34,0.62] | 0.49 ± 0.071 | [0.42,0.67] | 0.55 ± 0.062 | |
|  | MRD | [0.40,0.65] | 0.53 ± 0.065 | [0.38,0.65] | 0.52 ± 0.067 | |
| Bright Red | PRL | [0.42,0.65] | 0.54 ± 0.061 | [0.31,0.57] | 0.44 ± 0.066 | |
|  | LMCA | [0.41,0.68] | 0.54 ± 0.070 | [0.25,0.49] | 0.37 ± 0.060 | |
|  | Max_PC | [0.38,0.66] | 0.52 ± 0.074 | [0.34,0.63] | 0.48 ± 0.073 | |
|  | MCA | [0.39,0.65] | 0.52 ± 0.067 | [0.34,0.60] | 0.48 ± 0.069 | |
|  | MCD | [0.36,0.64] | 0.51 ± 0.070 | [0.40,0.67] | 0.53 ± 0.068 | |
|  | AC | [0.32,0.59] | 0.45 ± 0.070 | [0.35,0.64] | 0.50 ± 0.075 | |
|  | LMRD | [0.38,0.66] | 0.53 ± 0.069 | [0.32,0.58] | 0.45 ± 0.065 | |
|  | LMRV | [0.36,0.66] | 0.51 ± 0.075 | [0.39,0.64] | 0.52 ± 0.066 | |
|  | LMP | [0.39,0.64] | 0.52 ± 0.067 | [0.28,0.54] | 0.42 ± 0.067 | |
|  | LMRA | [0.36,0.61] | 0.49 ± 0.065 | [0.35,0.62] | 0.49 ± 0.068 | |
|  | MRA | [0.38,0.64] | 0.51 ± 0.064 | [0.40,0.64] | 0.52 ± 0.064 | |
|  | MCV | [0.35,0.61] | 0.48 ± 0.065 | [0.39,0.64] | 0.52 ± 0.065 | |
|  | MRV | [0.39,0.65] | 0.52 ± 0.068 | [0.42,0.69] | 0.56 ± 0.069 | |
|  | PRP | [0.36,0.63] | 0.50 ± 0.068 | [0.32,0.59] | 0.45 ± 0.068 | |
|  | LMCD | [0.38,0.64] | 0.51 ± 0.067 | [0.29,0.52] | 0.41 ± 0.061 | |
|  | LMCV | [0.37,0.63] | 0.51 ± 0.066 | [0.44,0.71] | 0.57 ± 0.070 | |
|  | MRD | [0.41,0.69] | 0.55 ± 0.070 | [0.49,0.76] | 0.63 ± 0.070 | |

*Data are resented as the Confidence Interval (CI), Mean ± Standard Deviation (SD) and AUC-ROC, using 2000 iterations, for each one of 34 trained models. Each row indicates one PLR parameter. Abbreviations of PLR parameters are described in Supplementary Method 1.*
